# The Applicability of Forest Therapy for Socially Isolated Young Adults: Parallel Changes in Psychological Distress and Mindfulness

**DOI:** 10.3390/ijerph23081078

**Published:** 2026-08-19

**Authors:** Yeji Yang, Ji-Eun Pyo, Yongjun Lee, Beom Lee, Chang-Hyou Kim, Jeong-Ho Choi, Jina Yu, Jisoo Lee, Kee-Hong Choi

**Affiliations:** 1 School of Psychology, Korea University, Seoul 02841, Republic of Korea; yaeji0917@korea.ac.kr (Y.Y.); ansgkr99@naver.com (Y.L.); lylayu852@naver.com (J.Y.); 2 KU Mind Health Institute, Korea University, Seoul 02841, Republic of Korea; pyofff@gmail.com (J.-E.P.); jisoo0105@korea.ac.kr (J.L.); 3 Forest Welfare R&D Center, Korea Forest Welfare Institute, Daejeon 35377, Republic of Korea; beom@fowi.or.kr (B.L.); uptake@fowi.or.kr (J.-H.C.)

**Keywords:** mindfulness, forest therapy, isolation, psychological distress, sustainable development goal

## Abstract

**Highlights:**

**Public health relevance—How does this work relate to a public health issue?**

- Social isolation among young adults is an emerging public health concern associated with elevated psychological distress and poorer mental health outcomes.
- This study evaluated the applicability of forest therapy as a nature-based intervention to reduce depression, anxiety, stress, and loneliness in socially isolated young adults.

**Public health significance—Why is this work of significance to public health?**

- A two-day forest therapy program was associated with reductions in psychological distress and improvements in mindfulness among socially isolated young adults.
- Improvements in nonreactivity, non-judging, and acting with awareness were most closely associated with reductions in psychological distress, highlighting potential mechanisms underlying the benefits of forest therapy.

**Public health implications—What are the key implications or messages for practitioners, policy makers and/or researchers in public health?**

- Forest therapy may serve as a promising community-based mental health intervention for socially isolated young adults, particularly when combined with mindfulness-enhancing activities.

**Abstract:**

This study examined the applicability of forest therapy in alleviating psychological distress among socially isolated young adults, with a particular focus on their associations with the mindfulness. A total of 159 socially isolated young adults were recruited from the community and participated in a two-day, one-night forest therapy program. Psychological distress (depression, anxiety, loneliness, and stress) and mindfulness were assessed before and after the program. Multilevel analyses were conducted to examine changes in psychological distress and mindfulness following participation, as well as whether the five mindfulness subcomponents (nonreactivity, observing, acting with awareness, describing, and non-judging) were associated with individual differences in changes in these outcomes. The results indicated reductions in depression, anxiety, stress, and loneliness, along with an increase in overall mindfulness after the program. Among the mindfulness subcomponents, reductions in psychological distress over time occurred most closely in parallel with changes in nonreactivity, non-judging, and acting with awareness, whereas the parallel was weaker for describing and observing. These findings suggest that forest therapy may be a promising intervention for reducing psychological distress among socially isolated young adults, and that incorporating activities designed to cultivate mindfulness skills—particularly nonreactivity, non-judging, and awareness—could further enhance its mental health benefits. As this study did not include a control group, randomized controlled trials are needed to confirm these effects.

## 1. Introduction

Social isolation refers to the insufficient quality and quantity of social relationships across individuals, groups, communities, and broader societal levels in which human interactions take place. Although closely related, social isolation and loneliness are distinct constructs. Loneliness is a multidimensional concept, with emotional loneliness referring to the absence of an attachment figure (i.e., a sense of isolation) and social loneliness indicating the lack of a social network that provides a sense of belonging or being part of a community [1]. A population-based study reported a 12.3% prevalence of social isolation among adults [2]. While the objective social network of young adults is the largest, the prevalence of loneliness and perceived social isolation is the highest across all age groups [3]. Social isolation is associated with psychological distress, such as depression and anxiety, and negative outcomes, such as deteriorated physical health and reduced quality of life, highlighting the need for active interventions [4,5,6]. Furthermore, the profound economic impact of social disconnection—evidenced by an annual loss of £2.5 billion for UK employers due to turnover and diminished productivity [7]—underscores that social isolation and loneliness is a critical public health challenge with a substantial national burden.

Nature-based therapy, which uses natural environments as an intervention, has been effective in improving mental and physical health. Forest therapy, a form of nature-based therapy, encompasses various therapeutic approaches such as spending time in forest environments or engaging in forest-based activities and enhances psychological and physiological health by reducing stress, improving mood, and boosting immune function [8]. Forest therapy has been shown to benefit several populations, particularly in alleviating depression, anxiety, and stress and in promoting overall well-being [9,10,11], with cultivating mindfulness emerging as a factor that significantly moderates these reductions [12]. Taken together, these findings suggest that forest therapy may be a useful approach for reducing psychological distress, and that actively promoting mindfulness within such programs could further enhance these benefits.

Forest therapy is a promising community-based intervention for socially isolated young adults. For instance, forest therapy resulted in significant changes in university students’ mood and stress responses compared with the control group, with notable reductions in negative emotions (e.g., tension, depression, and anger) and increased positive emotions (e.g., vitality) [13]. Another study found significant improvements in mental health symptoms, attachment and interpersonal relationships among adolescents and young adults after they participated in wilderness therapy [14]. Furthermore, engaging in forest therapy activities with others increases social connectedness and helps reduce social isolation and loneliness [15,16].

Various theories have been proposed to explain the mechanisms underlying nature-based therapies, including forest therapy. These include the biophilia hypothesis [17], attention restoration theory [18], stress recovery theory [19], the three-circle model of affect regulation [20], and enhanced immune functioning [21]. From a psychological perspective, the mechanisms of nature-based therapies appear to be particularly associated with attention. According to the attention restoration theory, an urban lifestyle demands a considerable amount of attentional resources, which leads to cognitive fatigue and stress, but nature creates a restorative environment that allows humans to reduce cognitive fatigue and has certain psychological benefits [18,22]. The stress recovery theory proposes that exposure to nature alleviates emotional and physiological stress and that these changes are accompanied by sustained attention and intake [19].

Attention is a key concept of mindfulness, which is considered an important construct in psychological interventions. Mindfulness is defined as “the awareness that emerges through paying attention on purpose, in the present moment, and nonjudgmentally to the unfolding of experience moment by moment” [23]. Recent studies have reported a significant increase in mindfulness following nature-based interventions [24,25], and mindfulness in nature-based therapies has been associated with improvements in depression, anxiety, and quality of life [26]. A meta-analysis of mindfulness-based interventions in natural outdoor settings indicated that the effects of nature-based mindfulness were more pronounced in wild or forest environments (Hedge’s g = 0.66) than in gardens and parks (Hedge’s g = 0.33), although the between-group difference did not reach statistical significance [27]. Additionally, forest therapy involves a combination of forest-based activities that extend beyond physical activity, often incorporating psychotherapy, meditation, and other activities that promote mental health [8]. After reviewing multiple studies, Racz et al. [28] argued that forest therapy and nature-based mindfulness are promising interventions for improving physical and mental health. They also advocated for the integration of these approaches to establish a foundation for social prescriptions that can support conventional healthcare and improve health outcomes. In this context, forest therapy may provide a useful framework for supporting mental health in community settings. Furthermore, recent research has progressed beyond examining the role of mindfulness in nature to identify its core components. Mindfulness is commonly conceptualized as comprising five facets: nonreactivity, observing, acting with awareness, describing, and nonjudging [29]. Previous studies suggest that these facets may differ in their associations with nature connectedness and psychological well-being [30,31,32,33]. However, little is known about how individual mindfulness facets are related to changes in psychological distress following forest therapy.

Most previous studies have been limited to cross-sectional designs, and the role of individual mindfulness components in alleviating psychological distress through nature-based interventions has been scarcely examined. Exploring changes in mindfulness facets during the intervention process is crucial for identifying focal areas in mindfulness-integrated therapies [34]. Based on the findings of previous studies, this study investigated whether forest therapy interventions for socially isolated young adults lead to meaningful changes in psychological distress, including depression, anxiety, stress, and loneliness. Additionally, the study examined the association between changes in mindfulness and changes in psychological distress, with a specific focus on the five subcomponents (i.e., nonreactivity, observing, acting with awareness, describing, and non-judging of experience) of mindfulness. The specific hypotheses of this study were as follows:

(1) Participants’ psychological distress (i.e., depression, anxiety, loneliness, and stress) decreases significantly after their participation in the forest therapy program.
(2) Participants’ mindfulness levels increase significantly following their participation in the forest therapy program.
(3) The reduction in psychological distress after participation in the forest therapy program occurred in parallel with changes in all five facets of mindfulness.

## 2. Materials and Methods

### 2.1. Participants

**Figure 1:**
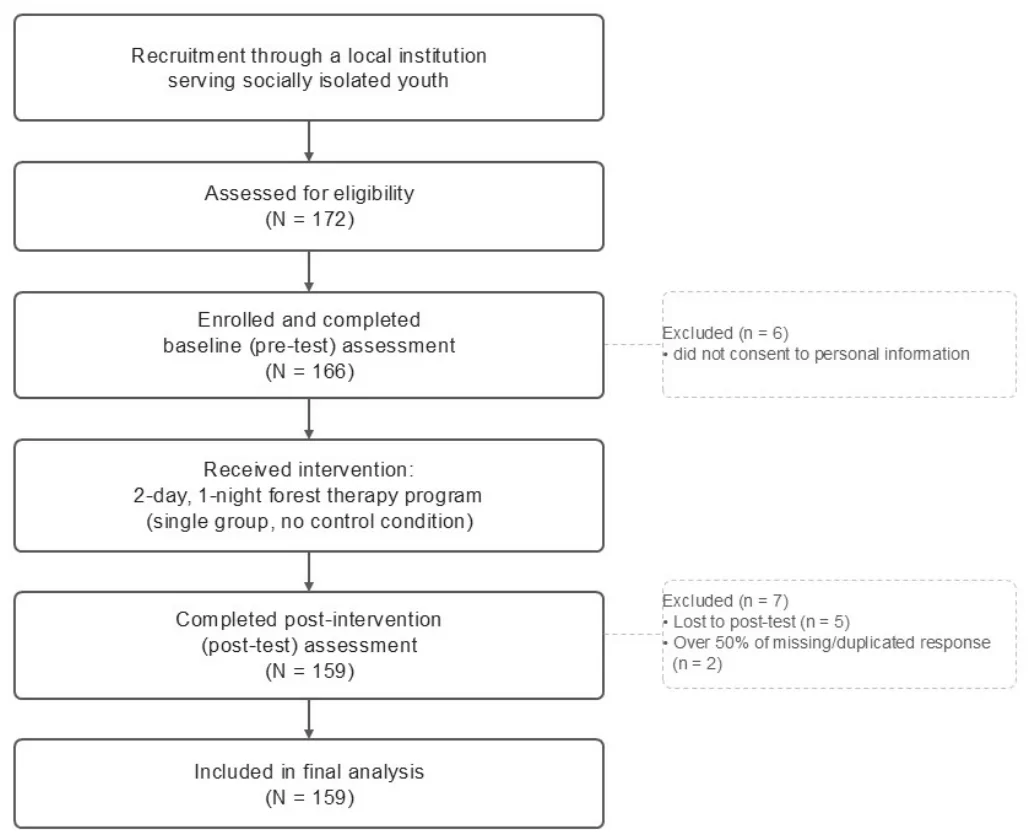
Flow diagram of the research.

This study adopted a pre-experimental design in which no control group was included, and all participants were assigned to the experimental group (Figure 1). Participants were young adults aged 19 to 39 years who had experienced social isolation. Although South Korea’s Framework Act on Youth defines youth as those aged 19 to 34, this upper limit was extended to 39 to reflect the broader age range increasingly adopted by local governments in response to population aging. Between August and November 2024, participants were recruited from local institutions for socially isolated youths who reported experiencing extreme isolation and seeking support for social reintegration. Individuals who felt isolated and desired psychological support were eligible to participate and no additional screening procedures were conducted. To best represent the community sample, the exclusion criteria were kept to a minimum, excluding only those with a state requiring immediate medical attention (e.g., severe suicidal ideation, psychosis, mania, or substance abuse) and physical disabilities that would hinder participation in the forest therapy program. These conditions were assessed through staff interviews and self-reports. Of the 172 participants in forest therapy, data from 6 who did not consent to personal information collection, 5 who missed post-assessments, and 2 with over 50% missing or duplicate responses were excluded, leaving a final dataset of 159 participants. The participants voluntarily participated in the study and provided consent for their participation and the use of their personal information. Before the study began, they were informed that they could discontinue their participation at any time. Pre- and post-intervention assessments were conducted on-site, immediately before and after the program. All research procedures were preregistered in the Clinical Research Information Service and approved in advance by the Local Institutional Review Board.

### 2.2. Measures

#### 2.2.1. Depressive Symptoms

The Mental Health Screening Tool for Depressive Disorders [35] was developed and validated in a Korean population for the early and accurate screening of patients with major depressive disorders and providing timely interventions in primary care settings. It includes 12 items measuring symptoms related to the diagnostic features of major depressive disorders presented in the Diagnostic and Statistical Manual of Mental Disorders, 5th edition, over the past two weeks and is rated on a 5-point Likert scale (0 = Never, 4 = Very much). The higher the score, the more severe the depression, and a score of 17 is the cutoff for major depressive disorder. The tool’s internal consistency was excellent as reported in a validation study conducted in Korea (Cronbach’s α = 0.94), and in this study, it was 0.93.

#### 2.2.2. Anxiety Symptoms

The Mental Health Screening Tool for Generalized Anxiety Disorders [36] was developed and validated with a Korean population to screen patients with generalized anxiety disorder early and accurately and provide timely intervention in primary care settings. It comprises 11 items assessing symptoms related to the diagnostic features of generalized anxiety disorder presented in the Diagnostic and Statistical Manual of Mental Disorders, 5th edition, over the past two weeks on a 5-point Likert scale (0 = not at all, 4 = always). The frequency of anxiety increases with higher scores, and a score of 15 is the cutoff for generalized anxiety disorder. A validation study conducted in Korea showed excellent internal consistency (Cronbach’s α = 0.94); in this study, it was 0.95.

#### 2.2.3. Loneliness

Loneliness was measured using the 8-item UCLA Loneliness Scale (UCLA-8) [37], a validated self-report measure of subjective loneliness. This scale is an indicator of the degree of subjective social isolation, and the deficiency of social contact is measured as the discrepancy between an individual’s desired and actual social contact on a 4-point Likert scale (0 = never, 3 = always). Items 3 and 6 are reverse-scored, with a higher total score indicating a higher level of loneliness. The scale demonstrated good internal consistency in the development study (Cronbach’s α = 0.84) [36], and the consistency was 0.84 in this study.

#### 2.2.4. Stress

The original Stress Response Scale comprising 39 items with subfactors of somatization, anger, and depression [38] was shortened and validated by Choi et al. [39]. This scale was developed for Korean adults, making it easy to implement with minimal resistance. The shortened version of the scale, consisting of 22 items, was used in this study to assess the extent to which individuals experience emotional, physical, cognitive, and behavioral symptoms in response to stressors using a 5-point Likert scale (0 = not at all, 4 = extremely). In a development study with Korean samples, it showed excellent internal consistency (Cronbach’s α = 0.97), and the value was 0.95 in this study.

#### 2.2.5. Mindfulness

The 15-item Five-Factor Mindfulness Questionnaire (FFMQ-15) [40] is a condensed and validated version of the Five-Factor Mindfulness Scale originally developed by Baer, Smith, Hopkins, Krietemeyer and Toney [29]. The FFMQ-15 assesses relatively stable individual differences in mindfulness across situations (i.e., trait mindfulness), rather than momentary mindful states at a specific moment in time (i.e., state mindfulness) [41]. The 15-item FFMQ includes 5 subscales—nonreactivity, observing, acting with awareness, describing, and non-judging of experience—each consisting of 3 items, totaling 15 items. The 15-item FFMQ allows for convenient mindfulness assessment in clinical settings. Items in the acting with awareness and non-judging of experience subscales are reverse-scored, with a higher total score indicating a higher level of mindfulness. The internal consistency of the 15-item FFMQ was reported as 0.76 in the validation study and 0.80 in this study. Reliability at the subscale level ranged from 0.64 (nonreactivity) to 0.82 (observing and describing), with awareness at 0.79 and nonjudging at 0.77. Corrected item-total correlations for the reverse-scored items across the two subscales ranged from 0.57 to 0.70, and reliability did not improve upon deletion of any item, suggesting no evidence of systematic misinterpretation of reverse-scored items in this sample.

### 2.3. Intervention

The forest therapy program was designed as a two-day, one-night experience comprising three distinct forest therapy activities: two in the afternoon of the first day and one in the morning of the second day. This program was specifically tailored for socially isolated young adults to facilitate psychological recovery, enhance physical vitality, and promote overall well-being through social interactions, and has been developed based on consultations with clinical psychologists, certified forest therapy instructors, and specialists in youth self-sufficiency support. Consistent with the theoretical framework described in the Introduction, the program combined nature exposure, mindfulness-based practices, group interaction, and light physical activity, as these components have been suggested to promote psychological restoration, stress reduction, and social connectedness in natural environments [8,18,19,27]. The program’s structure, formulated through expert consultation, can support socially isolated youths by fostering self-reflection in a natural environment and encouraging the formation of positive relationships with others, ultimately contributing to their emotional stability and reintegration into society. Participants were accommodated at the National Center for Forest Education (Daejeon, Republic of Korea) during the program.

The detailed schedule of the program was as follows. On the first day, a pre-assessment was conducted. The first was a group activity, titled “Direction Over Speed” and encouraged each group to engage in the forest as an enjoyable space, guiding them to walk slowly while reflecting on their current state. Through group missions, the participants were provided with opportunities for self-exploration, followed by a group discussion activity where they shared and reflected on their experiences. The second activity, “Rest in the Forest” involved sensory stimulation-based meditation, slow breathing exercises, and simple art therapy techniques, which helped stabilize the mind and body through cultural and therapeutic engagement with nature. In this activity, mindfulness techniques were employed during meditation or observation of natural objects to enhance engagement with nature. On the second day, the morning session, “Body and Mind Relaxation” comprised movement-based therapy to recharge energy levels and enhance the motivation for physical activity. Mindfulness techniques were employed through movement to foster attention to one’s body and mind. Upon completing all activities, a post-assessment was conducted. All sessions were conducted by nationally certified forest therapy instructors, who were specialists in forest therapy and have received training in forest meditation. They provided participants with information about forests and forest-based experiential activities and guided them to engage safely and fully in forest therapy. To minimize assessment bias, the certified forest therapy instructors who delivered the intervention were blinded to both the pre- and post-intervention assessments and were not involved in data collection. All assessments were administered by independent research staff.

### 2.4. Statistical Analysis

All statistical analyses were conducted by an analytical research team independent of the forest therapy providers. Descriptive statistical analyses were performed to summarize participants’ demographic characteristics and study variables. Subsequently, to test Hypotheses 1 and 2, a multilevel analysis (i.e., a hierarchical linear mixed model) was conducted to evaluate the statistically significant differences between the pre- and post-intervention scores, indicating the effects of forest therapy. This multilevel approach offers several advantages for longitudinal analyses. It allows for clustering at a higher level, enabling the simultaneous modeling of intra- and inter-individual changes [42,43]. In this study, a three-level model was used because of the heterogeneity of the data, which varied according to the dates of participation. Time was treated as a Level 1 parameter (within-subjects), whereas individuals and groups were analyzed as Level 2 and 3 parameters, respectively (between-subjects). Using a random-effects-only model, the intraclass correlation coefficient (ICC) was calculated to assess the proportion of variance at each level (i.e., time, individual, and group). Based on previous studies indicating that the levels of isolation vary by age, sex, educational level, and employment status [3], the demographic variables were controlled in the multilevel analysis. Cohen’s *d_rm_*, which corrects the standardized mean difference for the correlation between repeated measures, was calculated for each variable (depression, anxiety, loneliness, stress, and mindfulness) [44]. This effect size allows the resulting values to be interpreted according to the conventional thresholds for small, medium, and large effects (0.2, 0.5, and 0.8, respectively) proposed by Cohen [45] and later clarified by Lakens [44], and to be compared more directly with effect sizes reported in between-groups designs. To test Hypothesis 3, a difference-score correlation analysis was conducted. Difference scores were calculated by subtracting the pre-intervention scores from the post-intervention scores. Pearson’s correlation analysis was performed to examine the relationships between the difference scores of the variables. The Pearson correlation coefficient can be interpreted as small (*r* = 0.1), moderate (*r* = 0.3), or large (*r* = 0.5) [45]. Subsequently, a multilevel analysis was conducted treating each of the five mindfulness subscales as a parallel change variable for depression, anxiety, loneliness, and stress. Among the five mindfulness subscales, only those that showed significant results in the difference-score correlation analysis were included as parallel change variables. To minimize multicollinearity and facilitate interpretation, group-mean centering was applied [43]. Statistical analyses were conducted using R software version 4.3.2 [46]. R software was utilized to perform a descriptive statistical analysis, multilevel analysis, and Pearson correlation analysis using the “nlme” [47], “psych” [48] and “effsize” [49] packages.

## 3. Results

### 3.1. Demographic Characteristics

The baseline demographic characteristics of the participants are shown in Table 1. A total of 159 participants were included in this study, with a mean age of 27.94 years (age range = 19–39, standard deviation = 4.65). The participants were divided into 11 groups based on their participation dates. In terms of sex distribution, 59.7% were males and 40.3% were females, with a slightly higher proportion of males. Regarding employment status, the largest groups were unemployed (39.6%) and employed full time (37.1%). Most participants were single (93.1%), and more than half held a university bachelor’s degree. Regarding household size, single-person households were the most common (40.3%), followed by four-, three-, and two-person households.

### 3.2. Changes in Psychological Distress and Mindfulness Before and After Forest Therapy

The mean scores for depression, anxiety, mindfulness, loneliness, and stress pre- and post-intervention and the *p*-values from the multilevel analysis results are presented in Table 2 (the full results of the multilevel analysis are presented in Supplementary Table S1). The mean depression score decreased by 6.7 points from a baseline of 13.48 to a post-intervention score of 6.78, indicating a medium effect size (Cohen’s *d_rm_* = 0.63), which demonstrates a medium decrease in depressive symptoms. The ICC calculations revealed that 39.7% of the variance was attributed to the individual level (Level 2), 0.2% to the group level (Level 3), and approximately 60% to the time level (Level 1). The multilevel analysis revealed a significant decrease in depression scores over time (*B* = −6.781, *p* < 0.001).

The mean anxiety score decreased by 6.64 points from a baseline of 14.3 to a post-intervention score of 7.66, also reflecting a medium effect size (Cohen’s *d_rm_* = 0.61), indicating a medium reduction in anxiety symptoms. The ICC calculations revealed that 46.8% of the variance was attributed to the individual level (Level 2), 1.6% to the group level (Level 3), and 51.6% to the time level (Level 1). The multilevel analysis revealed a significant decrease in anxiety scores over time (*B* = −6.803, *p* < 0.001).

The mean stress score decreased by 12.97 points from a baseline of 28.77 to a post-intervention score of 15.8, indicating a medium effect size (Cohen’s *d_rm_* = 0.67), and a corresponding large reduction in stress. The ICC calculation revealed that 49.4% of the variance was attributed to the individual level (Level 2), 0.7% to the group level (Level 3), and nearly half to the time level (Level 1). The multilevel analysis revealed a significant decrease in stress scores over time (*B* = −13.074, *p* < 0.001). Sex also had a significant effect on stress (*B* = 6.237, *p* = 0.029), suggesting that baseline stress levels were higher in females (mean = 32.1) than in males (mean = 23.8). An additional multilevel analysis, including the time × sex interaction, found no significant effect, indicating no sex differences in the changes in stress over time (*p* = 0.199). Given the subgroup sizes (n = 94 females, n = 64 males), the study had 80% power to detect an interaction of *d* = 0.45 or larger; power to detect the observed effect size (*d* = 0.21) was low (26%), so this null result should be interpreted with caution.

The mean loneliness score decreased by 1.75 points from a baseline of 10.52 to a post-intervention score of 8.77, showing a small effect size (Cohen’s *d_rm_* = 0.33), which indicates a medium reduction in loneliness. The ICC calculations revealed that 67% of the variance was attributed to the individual level (Level 2), 9.2% to the group level (Level 3), and 23.8% to the time level (Level 1). The multilevel analysis revealed a significant decrease in loneliness scores over time (*B* = −1.850, *p* < 0.001).

Finally, the mean mindfulness score increased by 5.71 points from a baseline of 62.19 to a post-intervention score of 67.9, reflecting a small effect size (Cohen’s *d_rm_* = 0.44), indicating a medium improvement in mindfulness. The ICC calculations revealed that 58.4% of the variance was attributed to the individual level (Level 2), 0.7% to the group level (Level 3), and 40.9% to the time level (Level 1). The multilevel analysis revealed a significant increase in mindfulness scores over time (*B* = 5.602, *p* < 0.001). Employment status also had a significant effect on mindfulness (*B* = 1.808, *p* = 0.044), suggesting that baseline mindfulness levels were higher among unemployed individuals or students than among employed individuals. An additional multilevel analysis, including the time × employment interaction, found no significant effect, indicating no employment-related differences in the changes in stress over time (*p* = 0.463).

### 3.3. Difference-Score Correlation Analysis

The results of the difference-score correlation analysis are presented in Table 3. Changes in depression were significantly correlated with the mindfulness subscales of nonreactivity, observing, awareness, and non-judging. Among these, nonreactivity showed a moderate correlation (*r* = −0.362), whereas the other subscales demonstrated small correlations. Changes in loneliness were significantly correlated with the mindfulness subscales of nonreactivity, awareness, describing, and non-judging. Awareness (*r* = −0.369) and non-judging (*r* = −0.318) exhibited moderate correlations, whereas nonreactivity (*r* = −0.234) and describing (*r* = −0.173) showed small correlations. Changes in anxiety and stress were significantly correlated with all five mindfulness subscales. Anxiety demonstrated a moderate correlation with nonreactivity (*r* = −0.397) and small correlations with the other subscales. Similarly, stress showed a moderate correlation with nonreactivity (*r* = −0.386) and small correlations with the other subscales. All pairwise correlations in Table 3 remained significant (or nonsignificant) after applying the Benjamini–Hochberg FDR correction, indicating that the pattern of results was not an artifact of multiple testing.

### 3.4. Parallel Changes in Mindfulness and Psychological Distress

**Figure 2:**
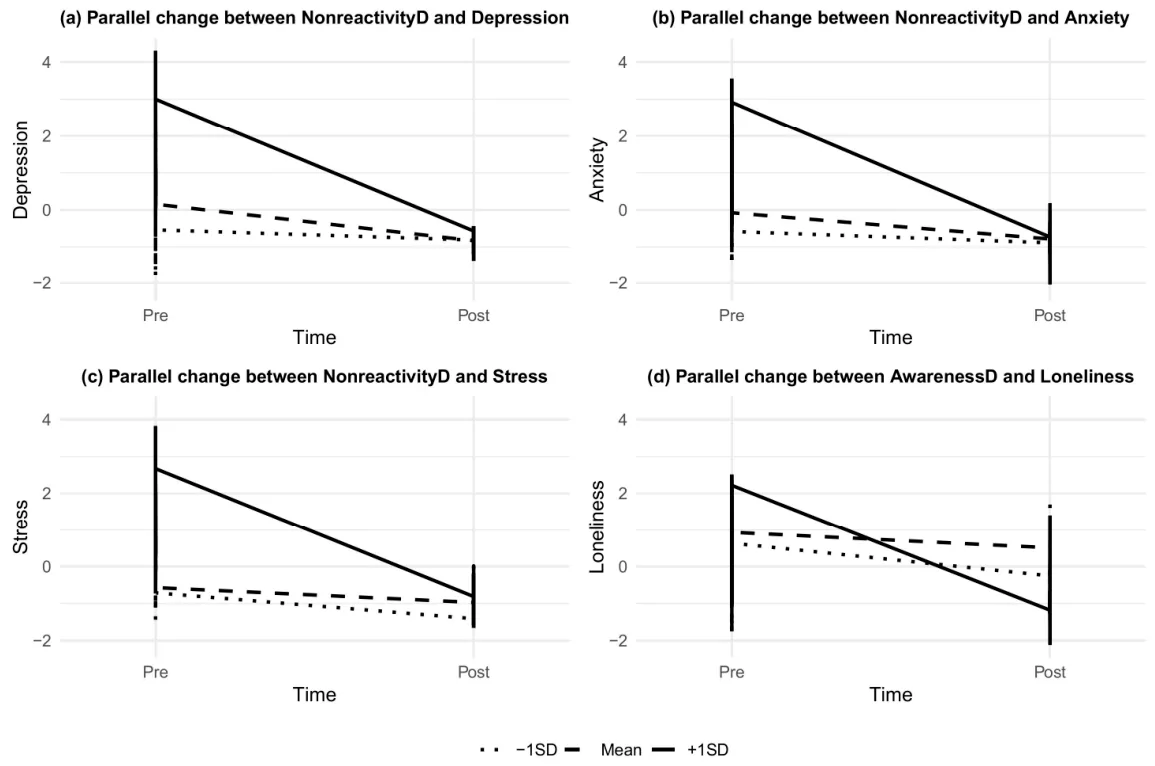
Parallel change between nonreactivity and (**a**) depression, (**b**) anxiety, and (**c**) stress, and between (**d**) awareness and loneliness. Note. To allow direct visual comparison of effect magnitudes across outcome variables measured on different scales, predicted values were standardized (z-scored) prior to plotting. NonreactivityD indicates the difference score of nonreactivity, and AwarenessD indicates the difference score of awareness. The dashed line represents the mean difference score in the mindfulness subscale, the solid line represents +1 standard deviation, and the dotted line represents −1 standard deviation.

The associations between changes in mindfulness subscales and changes in depression, anxiety, loneliness, and stress are presented in Figure 2 and Supplementary Tables S2–S5. For depression, the covariation between changes in four mindfulness subscales (nonreactivity, observing, awareness, and non-judging) and change in depression was examined, as these subscales showed significant correlations with depression change in the preliminary difference-score correlation analysis. Changes in all four subscales were significantly associated with the magnitude of pre–post change in depression following participation in the forest therapy program, indicating that greater increases in these facets of mindfulness paralleled greater reductions in depression. The coefficients representing this covariation were as follows: nonreactivity (*B* = −1.035, *p* < 0.001), non-judging (*B* = −0.803, *p* < 0.001), awareness (*B* = −0.639, *p* = 0.003), and observing (*B* = −0.562, *p* = 0.007).

For anxiety, the preliminary difference-score correlation analysis revealed significant correlations between changes in anxiety and all five mindfulness subscales. Accordingly, the covariation between changes in all subscales and change in anxiety was examined. Changes in all five mindfulness subscales were significantly associated with the magnitude of pre–post change in anxiety following participation in the forest therapy program, indicating that greater increases in these facets of mindfulness paralleled greater reductions in anxiety. The coefficients representing this covariation were as follows: nonreactivity (*B* = −1.060, *p* < 0.001), non-judging (*B* = −0.739, *p* < 0.001), observing (*B* = −0.647, *p* < 0.001), awareness (*B* = −0.561, *p* = 0.005), and describing (*B* = −0.522, *p* = 0.017).

Regarding stress, the preliminary difference-score correlation analysis demonstrated significant correlations between changes in stress and all five mindfulness subscales. Therefore, the covariation between changes in all subscales and change in stress was examined. Changes in all five mindfulness subscales were significantly associated with the magnitude of pre–post change in stress following participation in the forest therapy program, indicating that greater increases in these facets of mindfulness paralleled greater reductions in stress. The coefficients representing this covariation were as follows: nonreactivity (*B* = −1.835, *p* < 0.001), awareness (*B* = −1.336, *p* < 0.001), observing (*B* = −1.305, *p* < 0.001), non-judging (*B* = −1.224, *p* < 0.001), and describing (*B* = −1.061, *p* = 0.006).

For loneliness, the preliminary difference-score correlation analysis demonstrated significant correlations between changes in loneliness and four mindfulness subscales—Nonreactivity, Awareness, Describing, and Nonjudging. Therefore, the covariation between changes in these four subscales and change in loneliness was examined. Changes in all four mindfulness subscales were significantly associated with the magnitude of pre-post change in loneliness following participation in the forest therapy program, indicating that greater increases in these facets of mindfulness paralleled greater reductions in loneliness. The coefficients representing this covariation were as follows: awareness (*B* = −0.392, *p* < 0.001), nonjudging (*B* = −0.306, *p* < 0.001), nonreactivity (*B* = −0.250, *p* = 0.002), and describing (*B* = −0.186, *p* = 0.026).

## 4. Discussion

### 4.1. Main Findings

This study examined whether a forest therapy program was associated with changes in psychological distress and increased mindfulness in socially isolated young adults. Additionally, this study analyzed the role of the five facets of mindfulness in reducing psychological distress. Regarding Hypotheses 1 and 2, after participating in forest therapy, participants showed significant reductions in depression, anxiety, and stress with medium effect sizes, while loneliness decreased with a small effect size. Additionally, mindfulness increased with a small effect size. No demographic differences were observed in these changes, suggesting that the forest therapy program positively impacted mental health regardless of the participants’ sex and employment status. These findings are consistent with previous studies reporting improvements in depression, anxiety, stress, life satisfaction and well-being following forest therapy programs in various populations (e.g., office workers, university students, and patients) [13,50,51,52]. Meanwhile, the magnitude of change occurring over such a short period raises the possibility of regression to the mean. Baseline severity was in fact negatively correlated with change across all outcomes (*rs* = −0.37 to −0.72), with participants who started out more severe showing larger decreases. Given that participants were self-selected help-seekers and no control group was included, this possibility is difficult to rule out.

Interestingly, the calculation of the ICC in the multilevel model revealed that for loneliness and mindfulness, individual variance accounted for the largest proportion of the overall variability. This indicates that while changes occur in loneliness and mindfulness over time (e.g., with program participation), individual characteristics have a greater influence. For instance, some individuals may consistently experience higher levels of loneliness, whereas others may consistently experience lower levels. This finding suggests the need to consider individual differences in the level of loneliness and mindfulness interventions. When considering the smaller effect sizes for loneliness and mindfulness compared with depression, anxiety, and stress, changes in loneliness and mindfulness may not be substantial after a two-day, one-night forest therapy program. This difference may be partly explained by the multidimensional nature of loneliness, which encompasses both emotional loneliness and the lack of social network, making meaningful reductions in loneliness more difficult to achieve through a brief intervention alone. In addition, a meta-analysis of interventions targeting loneliness among young adults indicated that psychological interventions and social skills training demonstrated the most promising effect sizes [53]. This study suggested the need for professionally facilitated multi-session group interventions that incorporate psychotherapeutic elements to effectively address loneliness.

Considering mindfulness, the outcomes may vary depending on whether the focus is on state or trait mindfulness. This study used FFMQ to measure trait mindfulness, which may not exhibit significant changes following short-term interventions. However, research suggests that sustained increases in mindfulness through ongoing mindfulness practices can lead to greater long-term improvements in trait mindfulness and reductions in psychological distress [54]. Therefore, future studies should investigate whether incorporating mindfulness training into forest therapy programs leads to improvements in trait mindfulness and psychological outcomes.

Regarding Hypothesis 3, the analysis of the relationship between reductions in psychological distress and changes in mindfulness following the forest therapy program revealed that nonreactivity, non-judging, and acting with awareness showed the closest associations with the magnitude of distress reduction, whereas describing and observing showed weaker associations. These results should be cautiously interpreted with the possibility that a null correlation for a 3-item subscale in a sample of this size may simply reflect limited statistical power. Nevertheless, these findings align with previous research emphasizing the importance of non-judging and non-reactivity in mindful engagement in nature experiences and proposed heightened awareness of the surrounding environment as one of the underlying mechanisms [55]. Through mindful awareness, sensitivity to perceptual experiences in nature can be enhanced, while a non-judgmental and non-reactive stance may facilitate psychological restoration by regulating unproductive patterns of thought related to the experience [55]. In particular, nonreactivity showed the strongest association with reductions in depression, anxiety, and stress, whereas awareness had the greatest effect on reducing loneliness. Considering previous studies in which nonreactivity was a key mediator in improving emotional awareness and lability [56,57], it may function as an emotion regulation strategy in the nature engagement as well. Moreover, non-reactivity may reduce vulnerability to depression by buffering against situational rumination and negative biases, as well as by attenuating automatic emotional responses [58]. Additionally, cross-sectional studies have identified non-judgment as a significant factor correlated with well-being, depression, anxiety, and stress, highlighting its importance in mental health [59]. Adopting a non-judgmental and compassionate attitude toward one’s experiences may confer protective effects against negative affect, and nonjudging was also associated with lower levels of depression over the long term [60]. By contrast, awareness had the strongest correlation with loneliness, suggesting that acting with awareness may be particularly relevant to reducing loneliness. Recognizing and acting in the present moment may help stabilize negative emotional experiences such as loneliness by reducing emotional instability [61]. Also, it has been reported that participation in a mindfulness-based group intervention, designed with the intent to foster mindful awareness of one’s moment-to-moment experience, led to a significant reduction in loneliness [62]. Creswell et al. [62] suggested that psychological perceptions of social disconnection may constitute an important component of loneliness, and that mindfulness-based interventions targeting these perceptions could reduce perceived social threat or psychological distress while enhancing awareness of present-moment activities, thereby lowering the subjective experience of loneliness.

When each psychological distress variable was examined individually, all five mindfulness factors showed significant correlations between changes in the reduction in anxiety and stress during forest therapy. However, describing showed no significant association with changes in depression and observing showed no association with loneliness. Previous studies have similarly reported weaker or non-significant associations of describing and observing with psychological distress compared with other mindfulness facets [29,59,60,63]. However, these findings should be interpreted cautiously, as the lack of significant associations may also reflect limited statistical power or the short duration of the intervention. In the process of mindfulness, describing external and internal experiences may not necessarily be beneficial when it is associated with articulating concerns about possible negative future outcomes [64]. Individuals with little to no experience in mindfulness or meditation may observe their experiences in a maladaptive manner, such that the way they observe does not necessarily align with mindfulness [65]. Considering the relatively small sample size and the number of statistical tests conducted, the non-significant findings for describing and observing should be interpreted cautiously. Rather than suggesting that these facets function less adaptively than the others, the results may simply reflect insufficient statistical power to detect relatively small effects. Therefore, the present findings provide stronger support for the potential relevance of nonreactivity, non-judging, and acting with awareness, while the roles of describing and observing should be re-examined in larger studies.

### 4.2. Strengths, Limitations and Future Directions

This study has several strengths. First, by employing multilevel analysis, it enabled the simultaneous modeling of both intra- and inter-individual changes, leading to a more accurate estimation of psychological changes. Second, by examining the associations of specific factors in mindfulness with each psychological outcome, the study provides preliminary insights into which components may be emphasized in future interventions. By examining the associations of the changes in five components of mindfulness on depression, anxiety, stress, and loneliness, this research may help inform the development of future mindfulness-based forest therapy interventions targeting specific psychological symptoms. Future interventions can be designed to apply the most effective mindfulness techniques for specific target symptoms, thereby improving the cost-effectiveness of community-based programs. For instance, training in nonreactivity may be associated with greater improvements in depression, anxiety, and stress. This may involve breath-focused practices in which thoughts and emotions are observed as they arise and allowed to pass without judgment. In contrast, activities aimed at enhancing awareness may be associated with reductions in loneliness.

The limitations of this study were as follows. First, in terms of study design, the absence of a control group and follow-up assessments is a limitation. Although the primary aim of this study was to assess the applicability of forest therapy for socially isolated young adults and to examine the role of mindfulness, the absence of a control group limits the ability to draw causal inferences. In addition, the observed improvements cannot be attributed solely to the effects of forest therapy. Alternative explanations, including regression to the mean, natural symptom fluctuation, increased social interaction, and non-specific intervention effects, should also be considered. Therefore, future studies should employ randomized controlled trials (RCTs) to establish causal inferences regarding the effectiveness of forest therapy. Moreover, this study did not include follow-up assessments, making it unclear for how long the positive benefits of forest therapy persist. Future studies should therefore include follow-up assessments after participants return to their usual environments to determine the durability of the observed changes. Second, although the study targeted socially isolated youths, the absence of standardized screening tools may limit the representativeness of the sample. In this study, participants were recruited through a specialized institution providing services for socially isolated young adults and included individuals who subjectively reported feelings of isolation and a need for psychological support. Furthermore, participants were self-referred individuals seeking psychological support through a specialized institution. As a result, some participants may have enrolled during periods of heightened psychological distress, which may have contributed to larger observed changes over time through natural symptom fluctuation or regression to the mean. Therefore, the reported effect sizes should be interpreted cautiously until replicated in studies using standardized screening procedures and comparison groups. For future research, it is recommended to implement screening using validated measures of social isolation and psychological distress to ensure greater sample representativeness. Third, the sample size may not be sufficient to fully elucidate the treatment mechanisms. Using G*Power 3 [66] for a multiple regression analysis with a medium effect size and power of 0.95, the required sample size for this study was calculated to be 138 participants. However, a larger sample size is recommended to account for random effects at the individual and group levels [67]. Furthermore, given that individual differences in loneliness and mindfulness were greater than the time difference, increasing the sample size would be needed to validate additional subgroup analyses based on demographic characteristics. Relatedly, although the pattern of pairwise correlations in Table 3 held up after applying the Benjamini–Hochberg FDR correction, the sample is still fairly small relative to the number of correlations tested, particularly given the reliance on 3-item subscales. Whether facets such as observing show genuinely weaker associations with the outcome variables, or simply lacked the power to detect one, cannot be fully resolved with the current data. Finally, this study did not examine the role of factors other than the five facets of the FFMQ. While this study focused on five subscales of the validated and widely used FFMQ, prior research has indicated that improvements in acceptance may also contribute to reducing loneliness through mindfulness-based interventions [68]. Moreover, as the FFMQ is a measure of trait mindfulness, it may not have been well suited to capturing the more immediate, state-like shifts in mindfulness that a two-day intervention would be expected to produce. It is also possible that participants interpreted and responded to the FFMQ items with a state-like framing in mind—momentarily reflecting on their mindfulness during or immediately following the forest therapy session—rather than reporting on their general, dispositional tendencies as the instrument was originally intended to assess. This potential mismatch between the trait-oriented wording of the FFMQ and participants’ possibly state-oriented interpretation may have introduced additional measurement noise. Therefore, further research is needed that incorporates measures of state mindfulness alongside additional facets such as acceptance, decentering, and body awareness, to more fully capture the range of mindfulness-related mechanisms underlying the mental health benefits of forest therapy.

## 5. Conclusions

In summary, this study suggests that forest therapy appears to be an applicable intervention for alleviating psychological distress among socially isolated young adults in community settings. The findings also indicate that specific facets of trait mindfulness may be differentially associated with improvements in psychological outcomes following forest therapy. For the mental health of socially isolated youth, incorporating activities that explicitly train mindfulness skills, particularly nonreactivity, non-judging, and awareness, into forest therapy programs may be beneficial. Given the relatively modest improvements observed in loneliness, future studies should examine whether longer-term or repeated forest therapy programs tailored to individual characteristics yield greater benefits. From a practical perspective, these findings may help inform the development of community-based mental health programs for socially isolated young adults. Organizations that provide support services for socially isolated youth, as well as public health agencies and community welfare centers, may consider incorporating structured forest therapy and mindfulness-based activities as complementary components of existing psychosocial support programs. Overall, the present findings support the potential of forest therapy as a complementary non-pharmacological approach to promoting mental health among socially isolated young adults.

## Figures and Tables

**Table 1:** Baseline demographics of participants.

Baseline Demographics	N (%)
Sex	
Male	95 (59.7%)
Female	64 (40.3%)
Age, *M* (*SD*)	27.94 (4.65)
Employment	
Unemployed	63 (39.6%)
Student	24 (15.1%)
Homemaker	3 (1.9%)
Part-time Employed	10 (6.3%)
Full-time Employed	59 (37.1%)
Marital Status	
Never married	148 (93.1%)
Married	10 (6.3%)
Divorced	1 (0.6%)
Education	
High school (≤12 years)	50 (31.4%)
College Bachelor’s degree (≤14 years)	16 (10.1%)
University Bachelor’s degree (≤16 years)	87 (54.7%)
Higher Education (>16 years)	6 (3.8%)
Household size	
Single-person household	64 (40.3%)
Two-person households	22 (13.8%)
Three-person households	26 (16.4%)
Four-person households	33 (20.8%)
Five-person households	9 (5.7%)
More than six-person households	5 (3.1%)

**Table 2:** Mean scores, effect sizes (Cohen’s *d*), and the multilevel analysis results of five measures.

		Pre (*N =* 159)	Post (*N =* 159)	Cohen’s *d_rm_*	Multilevel Analysis		
						Estimates	*p*-Value
Psychological Distress							
MHS:D	Mean	13.48	6.78	0.63	Intercept	20.038	<0.001 ***
	(SD)	(11.91)	(8.3)		Time	−6.781	
MHS:A	Mean	14.3	7.66	0.61	Intercept	17.381	<0.001 ***
	(SD)	(11.69)	(9.4)		Time	−6.803	
SRI	Mean	28.77	15.8	0.67	Intercept	31.471	<0.001 ***
	(SD)	(20.51)	(18.14)		Time	−13.074	
ULS-8	Mean	10.52	8.77	0.33	Intercept	12.178	<0.001 ***
	(SD)	(5.56)	(5.31)		Time	−1.850	
Mindfulness							
FFMQ-SF (Total)	Mean	62.19	67.9	0.44	Intercept	50.623	<0.001 ***
	(SD)	(12.81)	(13.1)		Time	5.602	
Nonreactivity	Mean	11.36	12.65	0.36	Intercept	10.076	<0.001 ***
	(SD)	(3.59)	(3.60)		Time	1.282	
Observing	Mean	12.33	13.96	0.38	Intercept	10.686	<0.001 ***
	(SD)	(4.50)	(4.21)		Time	1.642	
Awareness	Mean	12.66	14.09	0.35	Intercept	11.233	<0.001 ***
	(SD)	(3.97)	(4.16)		Time	1.428	
Describing	Mean	12.52	13.32	0.20	Intercept	11.725	0.002 **
	(SD)	(4.07)	(3.92)		Time	0.790	
Nonjudging	Mean	13.33	13.88	0.12	Intercept	12.801	0.061
	(SD)	(4.46)	(4.27)		Time	0.532	

Note: The Estimates and *p*-values are from the results of the multilevel analysis (see Supplementary Table S1 for the full result). SD: standard deviation; MHS:D, Mental Health Screening Tool for Depressive Disorders; MHS:A, Mental Health Screening Tool for Anxiety Disorders; SRI, Stress Response Inventory; ULS-8, Short Form of the UCLA Loneliness Scale; FFMQ-SF, Five Facet Mindfulness Questionnaire Short Form. *** *p*-value < 0.001, ** *p*-value < 0.01.

**Table 3:** Results of the difference-score correlation analysis.

		1	2	3	4	5	6	7	8	9
Psychological Distress	1. MHS:D	-								
	2. MHS:A	0.685 **	-							
	3. SRI	0.556 **	0.585 **							
	4. ULS-8	0.403 **	0.353 **	0.434 **						
Mindfulness	5. Nonreactivity	−0.362 **	−0.397 **	−0.386 **	−0.234 **	-				
	6. Observing	−0.215 **	−0.269 **	−0.291 **	−0.151	0.458 **	-			
	7. Awareness	−0.220 **	−0.188 *	−0.265 **	−0.369 **	0.213 **	0.100	-		
	8. Describing	−0.088	−0.190 *	−0.211 **	−0.173 *	0.492 **	0.591 **	0.223 **	-	
	9. Nonjudging	−0.299 **	−0.295 **	−0.270 **	−0.318 **	0.264 **	0.103	0.369 **	0.057	-

Note. Each variable indicates a different score. * *p*-value < 0.05, ** *p*-value < 0.01. MHS: D, Mental Health Screening Tool for Depressive Disorders; MHS: A, Mental Health Screening Tool for Anxiety Disorders; SRI, Stress Response Inventory; ULS-8, Short Form of the UCLA Loneliness Scale.

## Data Availability

The data presented in this study are available on request from the corresponding author due to the ethical reasons.

## Supplementary Materials

The following supporting information can be downloaded at: https://www.mdpi.com/article/10.3390/ijerph23081078/s1, Table S1: The result of multilevel analysis of five measures; Table S2: Parallel change between mindfulness subscales and depression; Table S3: Parallel change between mindfulness subscales and anxiety; Table S4: Parallel change between mindfulness subscales and stress; Table S5: Parallel change between mindfulness subscales and loneliness.
