# Supplementary material for: The Applicability of Forest Therapy for Socially Isolated Young Adults: Parallel Changes in Psychological Distress and Mindfulness
Source: Int J Environ Res Public Health. 2026 Aug 19;23(8):1078. doi: 10.3390/ijerph23081078 (PMC13513809; doi:10.3390/ijerph23081078)
Supplement: Supplementary file 1 [file ijerph-23-01078-s001.zip › ijerph-4398923-supplementary.pdf]

Table S1. The result of multilevel analysis of five measures.

	Effects Estimate	95% CI		*SE*	*df*	*t*	*p*-value
		Lower	*Upper*				
**MHS:D**							
Intercept	20.038	13.215	26.860	3.454	157	5.801	<0.001 ***
Time	−6.781	−8.440	−5.121	0.840	157	−8.071	<0.001 ***
Sex	1.147	−1.529	3.823	1.354	143	0.847	0.398
Education	−0.195	−1.703	1.314	0.763	143	−0.255	0.799
Family	−0.266	−1.182	0.651	0.464	143	−0.573	0.568
Employment	0.341	-0.919	1.602	0.638	143	0.535	0.593
**MHS:A**							
Intercept	17.381	10.049	24.713	3.712	157	4.682	<0.001 ***
Time	−6.803	−8.358	−5.247	0.787	157	−8.640	<0.001 ***
Sex	2.509	−0.463	5.481	1.503	143	1.669	0.097
Education	0.553	−1.120	2.225	0.846	143	0.653	0.515
Family	−0.285	−1.298	0.728	0.512	143	−0.556	0.579
Employment	0.793	−0.611	2.196	0.710	143	1.117	0.266
**SRI**							
Intercept	31.471	18.251	44.691	6.693	156	4.702	<0.001 ***
Time	−13.074	−15.552	−10.596	1.255	156	−10.422	<0.001 ***
Sex	6.237	0.644	11.831	2.830	143	2.204	0.029 *
Education	1.966	−1.184	5.115	1.593	143	1.234	0.219
Family	−0.296	−2.211	1.619	0.969	143	−0.306	0.760
Employment	1.281	−1.358	3.921	1.335	143	0.959	0.339
**ULS-8**							
Intercept	12.178	8.196	16.160	2.016	156	6.041	<0.001 ***
Time	−1.850	−2.508	−1.192	0.333	156	−5.556	<0.001 ***
Sex	0.725	−0.906	2.355	0.825	143	0.879	0.381
Education	0.043	−0.873	0.959	0.463	143	0.092	0.927
Family	−0.545	−1.094	0.004	0.278	143	−1.964	0.052
Employment	0.516	−0.257	1.289	0.391	143	1.318	0.190
**FFMQ**							
Intercept	50.623	41.687	59.559	4.524	156	11.190	<0.001 ***
Time	5.602	3.805	7.399	0.910	156	6.158	<0.001 ***
Sex	−3.311	−7.040	0.419	1.887	143	−1.755	0.082
Education	0.960	−1.139	3.059	1.062	143	0.905	0.367
Family	0.659	−0.612	1.931	0.643	143	1.025	0.307
Employment	1.808	0.046	3.570	0.891	143	2.028	0.044 *

Note: *SE* = Standard Error. *df* = degree of freedom. *t* = t-value. * *p*-value < 0.05, ***p*-value < 0.01, ****p*-value < 0.001. MHS:D Mental Health Screening Tool for Depressive disorders; MHS:A Mental Health Screening Tool for Anxiety disorders; ULS-8 A short form of the UCLA Loneliness Scale; SRI Stress Response Inventory; FFMQ-SF Five Facet Mindfulness Questionnaire Short Form.

Table S2. Parallel change between mindfulness subscales and depression.

Predictors	Effects Estimate	95% CI		*SE*	*df*	*t*	*p*-value
		Lower	Upper				
**Nonreactivity**							
Intercept	20.111	13.316	26.906	3.440	156	5.846	<0.001 ***
Time	−6.807	−8.396	−5.218	0.804	156	−8.462	<0.001 ***
Nonreactivity	1.971	1.090	2.851	0.445	142	4.426	<0.001 ***
Sex	1.134	−1.558	3.825	1.361	142	0.833	0.406
Education	−0.181	−1.697	1.334	0.767	142	−0.237	0.813
Family	−0.256	−1.184	0.671	0.469	142	−0.546	0.586
Employment	0.323	−0.950	1.596	0.644	142	0.502	0.617
Time×Nonreactivity	−1.035	−1.456	−0.614	0.213	156	−4.856	<0.001 ***
**Observing**							
Intercept	19.642	13.012	26.272	3.357	156	5.852	<0.001 ***
Time	−6.714	−8.225	−5.203	0.765	156	−8.775	<0.001 ***
Observing	1.206	0.372	2.040	0.422	142	2.858	0.005 **
Sex	1.204	−1.477	3.884	1.356	142	0.888	0.376
Education	−0.157	−1.669	1.355	0.765	142	−0.206	0.838
Family	−0.225	−1.145	0.694	0.465	142	−0.484	0.629
Employment	0.343	−0.917	1.603	0.637	142	0.538	0.591
Time×Observing	−0.562	−0.967	−0.157	0.205	156	−2.742	0.007 **
**Awareness**							
Intercept	19.948	13.198	26.699	3.417	156	5.837	<0.001 ***
Time	−6.814	−8.477	−5.150	0.842	156	−8.090	<0.001 ***
Awareness	0.741	−0.141	1.622	0.446	142	1.661	0.099
Sex	0.965	−1.668	3.598	1.332	142	0.725	0.470
Education	−0.184	−1.666	1.299	0.750	142	−0.245	0.807
Family	−0.347	−1.248	0.554	0.456	142	−0.761	0.448
Employment	0.521	−0.727	1.769	0.631	142	0.825	0.411
Time×Awareness	−0.639	−1.058	−0.220	0.212	156	−3.010	0.003 **
**Nonjudging**							
Intercept	19.740	13.094	26.386	3.364	156	5.867	<0.001 ***
Time	−6.717	−8.190	−5.244	0.746	156	−9.010	<0.001 ***
Nonjudging	1.300	0.442	2.158	0.434	142	2.995	0.003 **
Sex	1.210	−1.477	3.898	1.360	142	0.890	0.375
Education	−0.201	−1.712	1.310	0.764	142	−0.263	0.793
Family	−0.217	−1.139	0.704	0.466	142	−0.466	0.642

Employment	0.342	−0.919	1.604	0.638	142	0.536	0.593
Time×Nonjudging	−0.803	−1.207	−0.399	0.204	156	−3.928	<0.001 ***

Note: SE = Standard Error. df = degree of freedom. t = t-value. **p*-value < 0.05, ***p*-value < 0.01, ****p*-value < 0.001.

Table S3. Parallel change between mindfulness subscales and anxiety.

Predictors	Effects Estimate	95% CI		*SE*	*df*	*t*	*p*-value
		Lower	Upper				
**Nonreactivity**							
Intercept	17.487	10.172	24.802	3.704	156	4.722	<0.001 ***
Time	−6.848	−8.335	−5.360	0.753	156	−9.093	<0.001 ***
Nonreactivity	1.899	1.089	2.710	0.410	142	4.631	<0.001 ***
Sex	2.506	−0.482	5.494	1.512	142	1.658	0.100
Education	0.553	−1.128	2.234	0.850	142	0.650	0.517
Family	−0.278	−1.304	0.748	0.519	142	−0.535	0.593
Employment	0.785	−0.630	2.201	0.716	142	1.097	0.275
Time×Nonreactivity	−1.060	−1.446	−0.675	0.195	156	−5.431	<0.001 ***
**Observing**							
Intercept	17.261	9.995	24.528	3.679	156	4.693	<0.001 ***
Time	−6.788	−8.260	−5.317	0.745	156	−9.112	<0.001 ***
Observing	1.187	0.415	1.960	0.391	142	3.038	0.003 **
Sex	2.527	−0.456	5.511	1.509	142	1.674	0.096
Education	0.541	−1.139	2.222	0.850	142	0.637	0.525
Family	−0.263	−1.283	0.756	0.516	142	−0.511	0.610
Employment	0.807	−0.599	2.213	0.711	142	1.135	0.258
Time×Observing	−0.647	−1.020	−0.274	0.189	156	−3.428	<0.001 ***
**Awareness**							
Intercept	17.208	9.898	24.518	3.701	156	4.650	<0.001 ***
Time	−6.841	−8.380	−5.301	0.779	156	−8.778	<0.001 ***
Awareness	0.728	−0.088	1.544	0.413	142	1.763	0.080
Sex	2.403	−0.562	5.368	1.500	142	1.602	0.111
Education	0.589	−1.079	2.257	0.844	142	0.698	0.486
Family	−0.316	−1.328	0.696	0.512	142	−0.617	0.538
Employment	0.907	−0.500	2.315	0.712	142	1.274	0.205
Time×Awareness	−0.561	−0.953	−0.169	0.198	156	−2.826	0.005 **
**Describing**							
Intercept	17.335	10.048	24.622	3.689	156	4.699	<0.001 ***
Time	−6.813	−8.325	−5.300	0.766	156	−8.897	<0.001 ***
Describing	1.122	0.244	2.000	0.444	142	2.527	0.013 *
Sex	2.515	−0.455	5.486	1.503	142	1.674	0.096
Education	0.607	−1.074	2.287	0.850	142	0.714	0.477

Family	−0.287	−1.301	0.727	0.513	142	−0.559	0.577
Employment	0.767	−0.636	2.169	0.710	142	1.081	0.282
Time×Describing	−0.522	−0.950	−0.094	0.217	156	−2.407	0.017 *
**Nonjudging**							
Intercept	17.098	9.798	24.397	3.696	156	4.627	<0.001 ***
Time	−6.804	−8.298	−5.309	0.757	156	−8.991	<0.001 ***
Nonjudging	1.094	0.304	1.885	0.400	142	2.737	0.007 **
Sex	2.611	−0.368	5.590	1.507	142	1.733	0.085
Education	0.599	−1.072	2.271	0.846	142	0.709	0.480
Family	−0.226	−1.241	0.789	0.513	142	−0.441	0.660
Employment	0.780	−0.623	2.182	0.710	142	1.099	0.274
Time×Nonjudging	−0.739	−1.115	−0.362	0.191	156	−3.873	<0.001 ***

Note: SE = Standard Error. df = degree of freedom. t = t-value. *p-value < 0.05, **p-value < 0.01, ***p-value < 0.001.

Table S4. Parallel change between mindfulness subscales and stress.

Predictors	Effects Estimate	95% CI		*SE*	*df*	*t*	*p*-value
		Lower	Upper				
**Nonreactivity**							
Intercept	31.410	18.419	44.400	6.576	155	4.776	<0.001 ***
Time	−12.979	−15.273	−10.684	1.161	155	−11.175	<0.001 ***
Nonreactivity	3.596	2.236	4.957	0.688	142	5.225	<0.001 ***
Sex	6.229	0.662	11.796	2.816	142	2.212	0.029 *
Education	2.045	−1.090	5.181	1.586	142	1.289	0.199
Family	−0.119	−2.041	1.804	0.972	142	−0.122	0.903
Employment	0.976	−1.655	3.606	1.331	142	0.733	0.465
Time×Nonreactivity	−1.835	−2.523	−1.147	0.348	155	−5.268	<0.001 ***
**Observing**							
Intercept	30.916	17.815	44.018	6.633	155	4.661	<0.001 ***
Time	−12.977	−15.358	−10.597	1.205	155	−10.768	<0.001 ***
Observing	2.508	1.214	3.802	0.655	142	3.831	<0.001 ***
Sex	6.546	0.960	12.133	2.826	142	2.317	0.022 *
Education	2.066	−1.084	5.216	1.593	142	1.297	0.197
Family	−0.208	−2.126	1.709	0.970	142	−0.215	0.830
Employment	1.167	−1.461	3.795	1.329	142	0.878	0.382
Time×Observing	−1.305	−1.970	−0.641	0.337	155	−3.879	<0.001 ***
**Awareness**							
Intercept	30.325	17.353	43.297	6.567	155	4.618	<0.001 ***
Time	−13.055	−15.443	−10.667	1.209	155	−10.801	<0.001 ***
Awareness	1.183	−0.213	2.578	0.706	142	1.675	0.096
Sex	5.818	0.368	11.269	2.757	142	2.110	0.037 *

Education	2.178	−0.888	5.244	1.551	142	1.404	0.162
Family	−0.273	−2.136	1.589	0.942	142	−0.290	0.772
Employment	1.661	−0.928	4.249	1.309	142	1.268	0.207
Time×Awareness	−1.336	−2.020	-0.652	0.346	155	−3.857	<0.001 ***
**Describing**							
Intercept	31.168	18.079	44.256	6.626	155	4.704	<0.001 ***
Time	−13.038	−15.467	−10.609	1.230	155	−10.604	<0.001 ***
Describing	2.342	0.867	3.817	0.746	142	3.138	0.002 **
Sex	6.246	0.679	11.813	2.816	142	2.218	0.028 *
Education	2.184	−0.969	5.337	1.595	142	1.369	0.173
Family	−0.316	−2.228	1.596	0.967	142	−0.327	0.744
Employment	1.166	−1.457	3.790	1.327	142	0.879	0.381
Time×Describing	−1.061	−1.814	-0.309	0.381	155	−2.786	0.006 **
**Nonjudging**							
Intercept	30.893	17.691	44.094	6.683	155	4.623	<0.001 ***
Time	−13.004	−15.397	−10.610	1.212	155	−10.732	<0.001 ***
Nonjudging	1.758	0.412	3.104	0.681	142	2.581	0.011 *
Sex	6.406	0.797	12.014	2.837	142	2.258	0.026 *
Education	2.059	−1.089	5.208	1.593	142	1.293	0.198
Family	−0.154	−2.074	1.765	0.971	142	−0.159	0.874
Employment	1.214	−1.425	3.854	1.335	142	0.910	0.365
Time×Nonjudging	−1.224	−1.892	-0.556	0.338	155	−3.620	<0.001 ***

Note: SE = Standard Error. df = degree of freedom. t = t-value. **p*-value < 0.05, ***p*-value < 0.01, ****p*-value < 0.001.

Table S5. Parallel change between mindfulness subscales and loneliness.

Predictors	Effects Estimate	95% CI		*SE*	*df*	*t*	*p*-value
		Lower	Upper				
**Nonreactivity**							
Intercept	12.195	8.203	16.187	2.021	155	6.034	<0.001 ***
Time	−1.850	−2.519	−1.180	0.339	155	−5.456	<0.001 ***
Nonreactivity	0.443	0.108	0.778	0.169	142	2.616	0.010 *
Sex	0.701	−0.935	2.338	0.828	142	0.848	0.398
Education	0.054	−0.864	0.972	0.464	142	0.117	0.907
Family	−0.521	−1.076	0.033	0.281	142	−1.859	0.065
Employment	0.487	−0.290	1.265	0.393	142	1.239	0.218
Time×Nonreactivity	−0.250	−0.405	−0.095	0.078	155	−3.192	0.002 **
**Awareness**							
Intercept	12.135	8.151	16.119	2.017	155	6.017	<0.001 ***
Time	−1.847	−2.541	−1.154	0.351	155	−5.260	<0.001 ***
Awareness	0.600	0.290	0.910	0.157	142	3.828	<0.001 ***

Sex	0.736	−0.895	2.366	0.825	142	0.892	0.374
Education	0.054	−0.862	0.970	0.463	142	0.116	0.908
Family	−0.527	−1.077	0.022	0.278	142	−1.896	0.060
Employment	0.507	−0.270	1.285	0.393	142	1.290	0.199
Time×Awareness	−0.392	−0.531	−0.252	0.070	155	−5.558	<0.001 ***
**Describing**							
Intercept	12.180	8.216	16.145	2.007	155	6.069	<0.001 ***
Time	−1.843	−2.478	−1.207	0.322	155	−5.731	<0.001 ***
Describing	0.353	0.003	0.703	0.177	142	1.993	0.048 *
Sex	0.719	−0.914	2.351	0.826	142	0.870	0.386
Education	0.058	-0.863	0.978	0.466	142	0.124	0.902
Family	−0.539	−1.088	0.011	0.278	142	−1.937	0.055
Employment	0.497	−0.278	1.272	0.392	142	1.268	0.207
Time×Describing	−0.186	−0.350	−0.023	0.083	155	−2.254	0.026 *
**Nonjudging**							
Intercept	12.129	8.170	16.088	2.004	155	6.052	<0.001 ***
Time	−1.833	−2.464	−1.201	0.320	155	−5.736	<0.001 ***
Nonjudging	0.471	0.161	0.780	0.156	142	3.009	0.003 **
Sex	0.742	−0.895	2.380	0.829	142	0.896	0.372
Education	0.048	−0.869	0.966	0.464	142	0.104	0.917
Family	−0.527	−1.077	0.024	0.279	142	−1.890	0.061
Employment	0.502	−0.272	1.276	0.392	142	1.281	0.202
Time×Nonjudging	−0.306	−0.446	−0.165	0.071	155	−4.294	<0.001 ***

Note: SE = Standard Error. df = degree of freedom. t = t-value. *p-value < 0.05, **p-value < 0.01, ***p-value < 0.001.
